# A Review Article: Access Recirculation Among End Stage Renal Disease Patients Undergoing Maintenance Hemodialysis

**DOI:** 10.5812/numonthly.6689

**Published:** 2013-03-30

**Authors:** Abbasali Zeraati, Seyed Seifollah Beladi Mousavi, Marzieh Beladi Mousavi

**Affiliations:** 1 Department of Nephrology, Imam Reza Hospital, Mashhad University of Medical Sciences, Mashhad, IR Iran; 2 Department of Internal Medicine, Faculty of Medicine, Jundishapour University of Medical Sciences, Ahvaz, IR Iran; 3 Department of Chemistry, Islamic Azad University, Omidiyeh Branch, Omidiyeh, IR Iran

**Keywords:** Hemodialysis Solutions, Arteriovenous Fistula, Kidney Diseases

## Abstract

**Background:**

The presence of arterio-venous (A-V) fistula recirculation among hemodialysis (HD) patients markedly decrease adequacy of dialysis.

**Objectives:**

The present article summarize some of observations about clinical significance, causes, the most common techniques for measurement, and main source of pitfall in calculation of access recirculation.

**Materials and Methods:**

A variety of literature sources such as PubMed, Current Content, Scopus, Embase, and Iranmedex; with key words such as inadequate dialysis and arterio-venous fistula access recirculation were used to collect current data. Manuscripts published in English language as full-text articles or as abstract form were included in our review study.

**Results:**

Any access recirculation among HD patients should be considered abnormal and if it presents prompt investigation should be performed for its causes. There are two most common techniques for accurate assessment of access recirculation: Urea (or chemical) and nonurea-based method by ultrasound dilution technique. The most common causes of access recirculation are the presence of high-grade venous stenosis, inadequate arterial blood flow rate, close proximity, or misdirection of arterial and venous needles placement by HD staff especially in new vascular accesses due to a lack of familiarity with the access anatomy.

**Conclusions:**

The presence of access recirculation among HD patients can lead to significant inadequate dialysis thereby resulting in reducing the survival of these patients. Therefore, periodic assessment of access recirculation should be performed in HD wards.

## 1. Background

The life expectancy of patients with end stage renal disease (ESRD) is catastrophic in developed and developing countries (1-4). As an example, the United States renal data system reported that the mean survival of patients with ESRD aged 40 to 44 is approximately 8 years, and it is only 4.5 years for those 60 to 64 years of age (1). The range of the expected remaining life span in developing countries is also poor. In a study from Iran, Beladi Mousavi et al. reported that one, three, and five-year survival of their patients under chronic hemodialysis (HD) is 89.2%, 69.2%, and 46.8%, respectively (3)

Some of the risk factors for decreased survival of patients with ESRD have been identified including cause of ESRD, method of renal replacement therapy, inadequate dialysis, comorbid disease, psychosocial factors, nutritional state, and etc. (5-11). It is well established that inadequate dialysis is an important contributor to lower overall survival among patients undergoing maintenance dialysis. Therefore, assessment of adequacy of dialysis is a central issue in the management of these patients (5, 12).

The causes of inadequate dialysis are the presence of arterio-venous (A-V) fistula access recirculation (AR), machine- and patient-specific dialysis variables such as inadequate machine calibration, low blood flow rates, hypotensive episodes that require changes in treatment, and short term duration of HD session such as the late initiation of dialysis by staff, early termination because of patient request or clinical events, blood leak, and etc. (13-20).

The presence of A-V fistula recirculation markedly decrease adequacy of dialysis, resulting in dialysis delivery being less than that prescribed. Therefore, periodic assessment of dialysis access recirculation has important diagnostic implications among these patients. However, it seems that most of the HD centers neglect or disregard making a periodic assessment of access recirculation among their patients.

## 2. Objectives

There is a paucity of literature about this issue and the present article summarizes some of the observations about clinical significance, causes, the most common techniques for measurement, and main source of pitfall in calculation of access recirculation.

## 3. Materials and Methods

A variety of sources were used to collect current data for this systematic review. PubMed, Current Content, Scopus, Embase, and Iranmedex search of published articles was performed with key words such as inadequate dialysis and arterio-venous (A-V) fistula access recirculation. Manuscripts published in English language from January 1989 up to November 2011, as full-text articles and as abstract forms about A-V fistula access recirculation were included in our review study, although unfortunately we did not specifically search conference proceedings.

### 3.1. Definition of Access Recirculation

Normally the rate of blood flow through an A-V access and especially AV grafts is about 1 liter per minute. During HD the blood pump of HD machine, which normally pumps out blood from the access into the dialyzer, usually is set to take a flow of 300-500 cc per minute. Because the demand of blood pump is less than the blood flow of A-V access, usually all of the blood coming into the blood pump is coming from the arterial side of access. Now, in some instances for example in a failing A-V access, flow through the access can decrease markedly to less than the rate of blood pump of HD machine, therefore some of the dialyzed blood leaving the dialyzer through the venous needle reenters the dialyzer through the arterial side to support the extracorporeal blood flow rate set by the blood pump. This phenomenon is named access recirculation (AR) (21).

### 3.2. Clinical Significance of Access Recirculation

Access recirculation is diagnosed when the blood solute concentration in arterial line is lower than that of systemic circulation, indicating that there has been mixing of dialyzed blood with undialyzed blood entering the dialyzer. In this phenomenon, by mixing already dialyzed with undialyzed blood, the urea concentration in the blood entering the dialyzer may be reduced by 10-40% or more. Therefore when AR occurred, solute concentration gradients across the dialysis membrane and as a result the rate of removal of solutes are reduced (21). It is also recommended that the presence of high degrees of access recirculation should be suspected when there is an inadequate reduction in the blood urea nitrogen (BUN) (22).

Therefore the presence of significant access recirculation among patients with ESRD under HD after long time can lead to significant inadequate dialysis and discrepancy between the amount of HD prescribed and the amount of HD delivered, thereby resulting in reducing the survival of these patients. Any access recirculation should be considered abnormal, and prompt investigation should be performed in order to discover its causes (21).

The periodic measurement of HD access recirculation is also important for other reasons. A high degree of access recirculation is one of the surrogate markers of A-V fistula inflow problems among HD patients and early detection and treatment of these problems improves long-term access patency rates (21, 23). Although the method of screening for early detection of A-V fistula stenosis varies widely and is largely dictated by local customs and expertise, it seems that measurement of HD access recirculation can be used as a screening tool for this issue. It is also suggested that fistulography should be performed in elevated levels of access recirculation to determine whether stenotic lesions are impairing access blood flow (21, 23). In contrast, some authors suggested that measurement of recirculation may have a large analytical error and it also may be a late predictor of access dysfunction in AV fistulae. Therefore, they suggested that Doppler ultrasound may be more useful despite its increased cost (21, 24). On the other hand, measurement of recirculation is not an ideal screening tool for access dysfunction in AV grafts (21).

### 3.3. The Measurement of Access Recirculation

There are two most common techniques for accurate assessment of access recirculation: Urea (or chemical) and nonurea-based method by ultrasound dilution technique (or dilutional-based method) (22-32).

In ultrasound dilution-based method, two reusable clip ultrasound sensors are attached to the venous and arterial blood line, which are linked to a computer and access blood flow is checked. Then dialyzer blood lines are reversed; the ultrafiltration is turned off, and at a known flow rate, 10 ml of isotonic saline is quickly injected into the venous line to dilute the blood. The velocity of the blood dilution as it passes through the blood lines is measured by ultrasonography (27-29).

In urea-based method, the degree of access recirculation is measured by comparing the systemic and dialyzer inlet blood urea concentration (BUC) from the following formula:

Percent recirculation = ([P - A] ÷ [P - V]) × 100

In the above formula P, A, and V refer to the systemic urea concentrations in the peripheral blood, blood entering the arterial line, and post-dialyzer venous circuit, respectively.

If there were no recirculation, urea concentration in blood entering the dialyzer (A) would be equal to the systemic urea concentration (P), and the above formula would have a value of zero. On the other hand, access recirculation exists whenever the BUC in the blood entering the arterial line is lower than that in the peripheral sample, indicating dialyzed blood reenter to the arterial line rather than returning to the systemic circulation (22).

### 3.4. The Main Pitfall in Calculation of Access Recirculation 

The main source of pitfall in the formula of urea-based method is determination of the systemic urea concentration (P in the above formula). There are multiple methods for determination of the systemic urea concentration. The blood sample of peripheral vein in the contralateral arm ("three-needle" method) has traditionally been used for this issue. This method should not be utilized because of unnecessary venipuncture and overestimation of access recirculation in an unpredictable manner due to arteriovenous and venovenous disequilibrium (24-33). In the other method, blood sample from a peripheral artery is used, but although the BUC obtained from a peripheral arterial blood eliminates the effects of both arteriovenous and venovenous disequilibrium, arterial puncture during hemodialysis is also not practical and therefore is not recommended.

Appropriate alternative to the peripheral vein or three-needle method and peripheral artery is the low blood flow technique ("two-needle" method). In this method access blood flow of HD is reduced to 50 mL/min and 15 to 30 s later the blood sample from the arterial blood line is obtained for assessment of systemic BUC concentration (34).

### 3.5. The Causes of Access Recirculation

The most common causes of access recirculation are the presence of high-grade venous stenosis, inadequate arterial blood flow rate, and improper needle placement by HD staff during HD (21, 25, 35, 36).

High-grade venous stenosis can restrict dialyzed blood venous outflow, thereby, sometimes leading to backflow of some dialyzed blood to the dialytic circuit through the arterial needle. Therefore in this situation blood entering the dialyzer through the arterial side will become diluted with blood that has just left the dialyzer and as a result, the effective clearance obtained in the course of a HD session is reduced.

Access recirculation can also be induced by inadequate arterial inflow when the A-V fistula blood flow rate is less than the blood pump of HD machine (36). In this setting, backflow of some dialyzed blood from the venous side of the access to the arterial side is necessary to support the extracorporeal blood flow rate set by the blood pump.

Close proximity and or misdirection of arterial and venous needles are another cause of backflow or recirculation in HD especially in new vascular accesses due to a lack of familiarity with the access anatomy. Unfortunately, the role of improper needle placement in recirculation is usually ignored, but there is some evidence that it is a common cause of A-V fistula recirculation in some HD centers. As an example, in a cross sectional study, Beladi Mousavi et al. measured the degree of recirculation with urea based on the two-needle technique method among 100 patients with A-V fistula who were on HD for more than 3 months, and reported that misdirection of needles is the most common cause of A-V fistula recirculation in their center (25). Schneditz et al. have also reported that misplacement of arterial and venous needles is a common source of A-V fistula recirculation, even after such placement had been previously recognized (35). Therefore especially among patients with a new vascular access, an access diagram that depicts the arterial and venous limbs, should be obtained from the surgeon who constructed the access to aid HD staffs for appropriate placement of arterial and venous needles. If access diagram is not available, the anatomy can be determined by temporarily occluding the mid-portion of the graft. The portion retaining a pulse is the arterial side of access and the other portion is the venous limb (21).

## 4. Discussion

The measurement of dialysis access recirculation among ESRD patients undergoing maintenance HD has important diagnostic implications. The presence of access recirculation should be suspected when there is an inadequate reduction in the BUC, as shown by the post-dialysis BUC exceeding 40 percent of the pre-dialysis BUC. Therefore high degrees of access recirculation in long term can lead to significant inadequate dialysis. It is well established that inadequate dialysis is an important contributor to lower overall survival among these patients.

It is also suggested that the presence of access recirculation is one of the surrogate markers of A-V fistula inflow problems among HD patient and early detection and treatment of these problems improves long-term access patency rates. Therefore, periodic assessment of access recirculation may have an important effect in the management of ESRD patients undergoing maintenance HD.

Two most widely used methods for accurate assessment of access recirculation are nonurea-based dilutional method - by use of ultrasound - and two-needle urea-based (or chemical) method. It is recommended that the three-needle peripheral vein method for measurement of access recirculation should not be used.

In urea-based method, AR can be calculated from this formula.

Percent recirculation = ([P - A] ÷ [P - V]) × 100

Any access recirculation should be considered abnormal and if it is exceeding 10% in the urea-based method or exceeding 5% in the nonurea-based dilutional method, prompt investigation should be performed for discovering its causes. It is also recommended that fistulography should be performed in elevated levels of access recirculation to determine whether stenotic lesions are impairing access blood flow.

The most common causes of access recirculation are the presence of high-grade venous stenosis, inadequate arterial blood flow rate when the A-V fistula blood flow rate is less than the blood pump of HD machine, and close proximity or misdirection of arterial and venous needles placement by HD staff, especially in new vascular accesses due to a lack of familiarity with the access anatomy.
